# Effects of *Piper hemmendorffii* (Piperales: Piperaceae) Essential Oil and Limonene on Modulation of Phase I Detoxifying Enzymes and Acetylcholinesterase Activity of *Rhipicephalus microplus* (Acari: Ixodidae)

**DOI:** 10.3390/pathogens15080862

**Published:** 2026-08-19

**Authors:** Adalberto Alves Pereira Filho, Vladimir Fazito do Vale, Caio Marcio de Oliveira Monteiro, Mayara Macêdo Barrozo, Daniel Sobreira Rodrigues, Lydia Fumiko Yamaguchi, Massuo Jorge Kato, Ricardo Nascimento Araujo

**Affiliations:** 1 Laboratório de Artrópodes Hematófagos, Departamento de Parasitologia/ICB, Universidade Federal de Minas Gerais, Av. Antônio Carlos 6627, Pampulha, Belo Horizonte 31270-901, MG, Brazil; 2 Grupo de Pesquisa em Triatomíneos, Instituto René Rachou, Fiocruz, Belo Horizonte 30190-009, MG, Brazil; vladimir_fazito@yahoo.com.br; 3 Instituto de Patologia Tropical e Saúde Pública, Universidade Federal de Goiás, Rua 235, s/n, Setor Leste Universitário, Goiânia 74605-050, GO, Brazil; caiosat@gmail.com (C.M.d.O.M.); mayarabarrozo@discente.ufg.br (M.M.B.); 4 Empresa de Pesquisa Agropecuária de Minas Gerais, EPAMIG Campo Experimental Santa Rita, Prudente de Morais 34701-970, MG, Brazil; dsrodrigues.epamig@gmail.com; 5 Instituto de Pesquisas Tecnológicas do Estado de São Paulo, NUTABES, Avenida Professor Almeida Prado, 532, Butantã, São Paulo 05508-901, SP, Brazil; lydiafumiko@ipt.br; 6 Laboratório de Química de Produtos Naturais, Departamento de Química Fundamental, Instituto de Química, Universidade de São Paulo, Avenida Professor Lineu Prestes, 748, Butantã, São Paulo 05508-000, SP, Brazil

**Keywords:** cattle tick, bioinputs, natural products, α-esterase, β-esterase, CYP450

## Abstract

*Rhipicephalus microplus* is a cattle ectoparasite responsible for economic losses in livestock systems. The present study aimed to evaluate the essential oil (EO) of *Piper hemmendorffii* and its major constituent, limonene, for their activity against *R. microplus* larvae. The EO of *P. hemmendorffii* and its major compound, limonene, were evaluated against pyrethroid-resistant *R. microplus* larvae using larval immersion bioassays, biochemical analyses of detoxification enzymes and acetylcholinesterase, and scanning electron microscopy (SEM). The EO showed higher larvicidal potency (LC_50_ = 10.46 mg/mL; 95% CI: 9.86–11.09) compared to limonene (LC_50_ = 31.62 mg/mL; 95% CI: 30.86–32.40). *Piper hemmendorffii* EO affected the following classes of enzymes investigated: α-Esterase (α-EST) activity increased at LC_25_ and LC_50_, reaching its highest level at LC_50_, whereas β-esterase (β-EST) activity increased only at LC_25_. Cytochrome P450 (CYP450)-associated heme content activity showed a significant increase exclusively at LC_50_, suggesting a response of the surviving larvae to EO exposure, although this increase does not by itself demonstrate the activation of detoxification pathways. In contrast, limonene did not significantly affect the activity of the three evaluated enzymes. The inhibition of AChE by the EO suggests a possible effect on the cholinergic system, which may be associated with neurotoxic activity, whereas limonene had no significant effect. The absence of detectable ultrastructural changes by SEM suggests that treatment with either EO or limonene did not induce evident external morphological damage to the larval cuticle under the evaluated conditions. EO toxicity seems to be associated with changes in detoxification enzyme and AChE activities, suggesting the involvement of these biochemical pathways in larval response.

## 1. Introduction

*Rhipicephalus* (*Boophilus*) *microplus* Canestrini, 1888 is a monoxenous tick species whose parasitism, from larva to adult, is completed within approximately 20–25 days [1,2]. Commonly known as the cattle tick, it is considered one of the most significant ectoparasites affecting cattle worldwide. Its economic importance is primarily attributed to its hematophagous behavior, which results in substantial blood loss and reduced productivity in infested animals [3,4].

Given the economic impact of *R. microplus*, control strategies are needed. In this context, essential oils (EOs) and their bioactive constituents represent promising alternatives and warrant further investigation as potential acaricidal agents [5]. In Brazil, several studies have demonstrated the acaricidal potential of plant-derived products against *R. microplus*, including EOs from *Baccharis dracunculifolia* DC., *Lippia triplinervis* Gardner and *Syzygium aromaticum* (L.) Merr. & L.M. Perry, highlighting the relevance of botanical compounds as alternatives for tick control [6,7,8]. Species of the genus *Piper*, the largest in the Piperaceae family with approximately 2000 pantropical species, have attracted attention for their phytochemical diversity and reported acaricidal activity [9,10].

In the search for alternative acaricidal agents against *R. microplus*, several studies have reported acaricidal activity in species of the genus *Piper*, including *P. aduncum* L. [11], *P. tuberculatum* Jacq. [12], *P. nigrum* L. and *P. longum* L. [13]. *Piper hemmendorffii* C. DC. is a glabrous shrub distributed across several regions of Brazil, including the northeast (Bahia), central west (Mato Grosso do Sul), southeast (Espírito Santo, Minas Gerais, São Paulo), and south (Paraná) [14]. The leaves are lanceolate to oblong, with smooth surfaces, conspicuous glands, and acuminate to acute apices, and the fruits are laterally flattened, oblong, with truncated apices, and devoid of styles and trichomes [15]. To date, no studies have been reported evaluating its activity against *R. microplus.* Moreover, the selection of *P. hemmendorffii* was based on the chemical diversity and biological potential of the genus *Piper*, which includes species rich in specialized metabolites, such as phenylpropanoids, amides, and terpenoids, with reported pesticidal activities. Additionally, the documented acaricidal activity described above of *Piper* species supports the investigation of *P. hemmendorffii* as a potential source of bioactive compounds.

The detoxification of insecticidal and acaricidal compounds constitutes a major mechanism underlying the development of resistance in arthropods. This process often involves the upregulation of detoxification-related genes or increased catalytic activity of enzymes such as esterases (ESTs) and cytochrome P450 (CYP450), which are involved in phase I xenobiotic detoxification [16,17]. Therefore, evaluating these enzymatic markers not only helps us to understand how ticks respond to xenobiotic exposure but also provides valuable information about the potential mechanisms of action of candidate acaricides and their likelihood of inducing detoxification responses associated with resistance.

Acetylcholinesterase (AChE) is an additional target enzyme to be evaluated in the search for novel acaricidal compounds [18,19]. Within the cholinergic system of ticks, AChE performs its classical neural function by hydrolyzing acetylcholine in the synaptic cleft, thereby terminating nerve signal transmission [20]. The inhibition of AChE represents one of the main toxicological mechanisms of organophosphates and carbamates. However, mutations in the *AChE* gene can give rise to tick populations resistant to these insecticides by altering the enzyme’s structure, thus preventing effective inhibition [21].

This study aimed to evaluate the larvicidal activity of the essential oil (EO) of *P. hemmendorffii* and its major constituent, limonene, against *R. microplus* larvae by determining lethal concentrations; evaluating biochemical responses associated with exposure, particularly alterations in detoxification enzymes and acetylcholinesterase activity; and examining the larval cuticle for possible ultrastructural alterations using scanning electron microscopy (SEM).

## 2. Materials and Methods

### 2.1. Plant Material, Identification, and Essential Oil Extraction

The leaves of *P. hemmendorffii* C. DC. were collected in February 2024, during their fruiting stage in the period of January to June 2024 at the Institute of Chemistry, University of São Paulo (USP) (46° 43′32″ W; 23° 33′ 54″ S). A specimen of the plant was deposited at the Herbarium of USP for identification made by Dr. Eric Tepe and cataloged under registration (Kato-1086). Specimen collection was carried out under permission number #59161-1 from SISBIO (Sistema de Autorização e Informação em Biodiversidade).

The EO was extracted from fresh leaves submitted to hydrodistillation in a Clevenger-type apparatus for 4 h, at 95 °C, using 500 g of fresh leaves and 500 mL of distilled water, resulting in an EO yield of 0.86% [22,23]. The EO was collected and dried with anhydrous sodium sulfate and stored in amber bottles in a refrigerator at 4 °C until the experiments and analysis by gas chromatography coupled to mass spectrometry (GC-MS). Samples were analyzed on a Shimadzu GCMS-QP2010 equipped with an HP-5ms column (30 m × 0.25 mm ID × 0.25 μm; Agilent Technologies, Inc., Santa Clara, CA, USA). Helium was used as the carrier gas (1.55 mL min^−1^), and 1 μL of each sample was injected at 250 °C (split 1:20). The detector operated at 260 °C with electron impact ionization (70 eV), scanning *m*/*z* 35–400 at 2500 spectra s^−1^. The oven was held at 40 °C for 2 min, ramped at 5 °C min^−1^ to 260 °C, and held for 2 min. Volatile compounds were identified using the extracted ion chromatograms of three reference ions and quantified by the peak area of the most abundant ion using a custom method in GC-MS Postrun Analysis software (GCMS solution Version 4.45; Shimadzu Corporation, Kyoto, Japan) described previously [24]. The major compound from EO *P. hemmendorffii* characterized by GC-MS and used in this study was limonene. Reactive-grade limonene was obtained from Sigma-Aldrich (St. Louis, MO, USA; product no. 218367).

### 2.2. Tick Collection

Fully engorged *R. microplus* females were collected from two artificially infested calves at the Santa Rita Experimental Field (Campo Experimental Santa Rita—CESR) of the Agricultural Research Corporation of Minas Gerais (Empresa de Pesquisa Agropecuária de Minas Gerais—EPAMIG), located in the municipality of Prudente de Morais, Minas Gerais, Brazil (44°09′11″ W; 19°27′15″ S). Approximately 500 ticks were collected from each animal, and ticks from both calves were combined to form pooled samples for subsequent analyses. The eggs were collected and transferred to 20 mL conical-bottom tubes sealed with cotton plugs to allow for air circulation and moisture exchange. The samples were maintained in a biochemical oxygen demand (BOD) incubator at a controlled temperature of 28 ± 2 °C and relative humidity of 90 ± 5% until larval hatching at the Laboratory of Hematophagous Arthropods (Laboratório de Artrópodes Hematófagos—LAH), following the recommendations of the Ethics Committee on Animal Use (Comissão de Ética no Uso de Animais—CEUA/EPAMIG) under protocol number 10/2023. The collection of the specimens was carried out under authorization granted by SISBIO (Sistema de Autorização e Informação em Biodiversidade), protocol A36A41C. Subsequently, larvae aged 20 to 40 days post-hatching were maintained under the same temperature and humidity conditions described above. Under these conditions, potential age-related variations in the basal activity of detoxification enzymes, such as esterases, are expected to be minimized. Furthermore, larvae were randomly allocated among the experimental groups, reducing the possibility of age-related bias and ensuring comparability among treatments. This tick population was characterized as resistant (resistance level I) to the synthetic pyrethroid cypermethrin. Resistance was determined using the larval immersion test with cypermethrin, according to the guidelines established by the Technical Document Guidelines for sustainable tick control and Acaricide Resistance [25] and as previously described [26].

### 2.3. Larval Immersion Test

The larval immersion test (LIT) was carried out according to the method described by Klafle et al., 2006 [27], with some modifications as detailed below. *P. hemmendorffii* EO was tested at concentrations of 3, 5, 8, 10, 13, 16, 20, and 24 mg/mL, and limonene was tested at concentrations of 15, 20, 25, 30, 35, 40, 45, and 50 mg/mL on *R. microplus*. All concentrations were prepared using a solution of 1% acetone and 0.02% Triton X-100 in a final volume of 1 mL. The LIT was conducted across five independent experiments, each consisting of two replicates. The control group received a treatment with a solvent solution containing 1% acetone and 0.02% Triton X-100. For each concentration, approximately 100–150 larvae were immersed for 10 min. The tubes were then immediately sealed, briefly agitated for 5 s, and left to rest for 10 min. Subsequently, the larvae were allowed to dry on filter paper before being transferred to a dry filter paper packet (8.5 × 7.5 cm) which was then secured with plastic clips. Packages were maintained in a BOD incubator at 27 ± 1 °C and ≥80% relative humidity for 24 h. After incubation, larvae were counted using a vacuum suction apparatus (Primar, model 141, São Paulo, SP, Brazil), and those unresponsive to CO_2_ stimulation (approximately 10–20 s) were classified as dead. Mortality was expressed as a percentage, calculated as the number of dead larvae divided by the total estimated number of larvae initially placed in each concentration.

### 2.4. Quantitative Analysis of Detoxification Enzymes and Acetylcholinesterase Activity

#### 2.4.1. Treatment and Processing of Larvae

Larvae of *R. microplus* were treated with LC_5_, LC_25_, LC_50_, and LC_75_ of EO *P. hemmendorffii* or limonene. Approximately 1000 larvae were added to microcentrifuge tubes containing the lethal concentrations of EO *P. hemmendorffii* or limonene for larval treatment. The tubes were immediately closed, shaken for 5 s, and left to rest for 10 min. After this period, the larvae were dried on filter paper and transferred to filter paper packages (10 × 6 cm) which were then closed with clips. The packages were placed in a BOD incubator at 27 °C and RH ≥ 80% for 24 h, and larvae that survived the treatments were collected for further processing.

Surviving larvae were immediately macerated in PBS buffer (NaCl 136.8 mM; KCl 2.7 mM; Na_2_HPO_4_ 4.76 mM; KH_2_PO_4_ 1.76 mM) containing 0.1% Triton™ X-100 reduced (Sigma-Aldrich, St. Louis, MO, USA; Cat. No. X100) and centrifuged (12,000× *g*, 10 min at 4 °C). The supernatant was removed and transferred to another microcentrifuge tube, where a mixture containing protease inhibitors at a final concentration of 1 µM Pepstatin A and 1.5 mM EDTA was added, and tubes were kept at −80 °C until the measurement of enzymatic activity. Total protein in all extracts was measured using Pierce™ BCA Protein Assay Kits Manufacturer: Thermo Scientific, Waltham, MA, USA; Cat. No. A55864 with BSA as the standard, according to the manufacturer’s protocol, at an absorbance of 562 nm. The diluent was used as the negative control.

The diluent, composed of 1% acetone and 0.02% Triton™ X-100, was used as the negative control and was also used to prepare the concentrations of each treatment. Blank samples were obtained by substituting the 10 µL aliquots of each sample with 10 µL of PBS containing 0.1% Triton X-100 reduced. Positive control inhibitors were not included because the objective of the enzymatic assays was to compare treatment-induced changes in enzyme activity relative to the vehicle control, rather than to validate enzyme inhibition using reference compounds. During enzymatic activity assays, the absorbance of each test sample was normalized by subtracting the mean blank value. All experiments were conducted in duplicate (technical replicates), and five independent assay (biological replicates) runs were carried out for statistical evaluation.

#### 2.4.2. Assay of Activity of Mixed-Function Oxidases

Mixed-function oxidases were quantified indirectly following the method of [28]. Assays were performed in BIOFLOAT™ 96-well plates using larval protein extracts, while blank wells received PBS containing 0.1% Triton X-100 instead of extract. Cytochrome C standards (0.00031, 0.000625, 0.00125, 0.0025, 0.005 and 0.01 mg/mL) were used to generate the calibration curve.

Each well received 60 μL of 90 mM potassium phosphate buffer (pH 7.2), 200 μL of 3,3′,5,5′-tetramethyl-benzidine dihydrochloride (TMBZ) solution 0.05%, and 25 μL of 3% H_2_O_2_, resulting in a final volume of 305 μL. Plates were incubated for 90 min at room temperature in the dark, and absorbance was measured at 650 nm. The assay provides an indirect estimate of CYP450-associated heme/mixed-function oxidase content, expressed as ng CytC/µg protein.

#### 2.4.3. Esterase Activity Assay

Esterase activity was determined using α-naphthyl acetate and β-naphthyl acetate as substrates for α- and β-esterase, respectively, following the method described by Van Asperen (1962) [29]. Each well of a BIOFLOAT™ 96-well plate contained 200 μL of reaction mixture composed of 10 μL of larval protein extract and 190 μL of solution containing 40 mM sodium phosphate buffer (pH 7.2), distilled water, and 30 mM α- or β-naphthyl acetate.

For the standard curve, α- or β-naphthol solutions (0, 0.001, 0.0025, 0.005, 0.0075, 0.01, 0.015, and 0.02 µmol) were prepared in acetone and added to wells containing phosphate buffer, distilled water, and 0.1% Triton X-100 in PBS. Plates were incubated for 30 min at 30 °C, followed by the addition of 50 μL staining reagent (3.4% SDS and 0.3% Fast Blue) and a further 5 min incubation at 30 °C. Absorbance was measured at 570 nm using a Versamax ELISA plate reader (Molecular Devices). One unit (U) of enzymatic activity is defined as the amount of enzyme that produces 1 µmol of product per minute, and esterase activity was expressed as µU/µg protein.

#### 2.4.4. Acetylcholinesterase Activity Assay

Acetylcholinesterase activity was determined according to Li et al. (2005) [30], with modifications. In BIOFLOAT™ 96-well plates, 10 μL of larval protein extract was mixed with 190 μL reaction solution containing acetylthiocholine iodide, 100 mM sodium phosphate buffer pH 7.8, and distilled water, followed by incubation at 30 °C for 60 min. After incubation, 50 μL of staining reagent (2% Sodium Dodecyl Sulfate (SDS), 6 mM 5,5′-Dithiobis 2-nitrobenzoic acid DTNB, and 10 mM sodium phosphate buffer, pH 7.8) was added, and plates were incubated for an additional 5 min at 30 °C. Absorbance was measured at 410 nm using a VersaMax ELISA Microplate Reader (Molecular Devices, Versamax, Biloxi, MS, USA). The mean absorbance was corrected using the corresponding blank, expressed per minute according to the reaction period, and normalized to the protein content, yielding AChE activity as Abs/min/μg protein. Values were multiplied by 10^3^ solely for graphical presentation.

### 2.5. Scanning Electron Microscopy (SEM)

Scanning electron microscopy (SEM) was performed to investigate whether the larval mortality induced by the treatments was associated with ultrastructural alterations in the cuticle. To this end, live larvae of *R. microplus* were grouped according to the following treatments and maintained for 24 h: vehicle control (1% acetone and 0.02% Triton X-100), *P. hemmendorffii* EO at LC_50_ and LC_75_ concentrations, and limonene at LC_50_ and LC_75_ concentrations. After 24 h of exposure, ten larvae from each experimental group were fixed in a solution containing 2% paraformaldehyde and 2% glutaraldehyde for a period of 21 days. After 15 days, the fixative was replaced and maintained until the completion of the fixation period. The samples were then washed four times in phosphate buffer for 15 min each and subsequently stored in 70% ethanol for 24 h.

Dehydration was performed in an ascending ethanol series (80%, 90%, 95%, and 100%), with samples remaining for 30 min at each step. Following this procedure, the samples were transferred to 1.5 mL microtubes and placed in Falcon tubes containing absolute ethanol. Chemical drying was performed using hexamethyldisilazane (HMDS) for 5 min, followed by the removal of excess reagent and evaporation at room temperature. After drying, the specimens were mounted on cylindrical metallic stubs using conductive double-sided copper tape and coated with gold using a deposition system (Desk V, Denton Vacuum LLC, Moorestown, NJ, USA). Ultrastructural analyses were conducted using a scanning electron microscope (JSM-6610, JEOL, Tokyo, Japan) equipped with an EDS system Thermo Scientific NSS Spectral Imaging (Thermo Fisher Scientific, Waltham, MA, USA), operating at 8 kV.

### 2.6. Statistical Analysis

The data were organized in spreadsheets using Microsoft Excel 2007. To calculate the LCs, the data were initially transformed to log(X) and normalized, and then nonlinear regression was conducted to get LC_50_ using GraphPad Prism 7.0 software (GraphPad Inc., San Diego, CA, USA). LC_5_, LC_25_, and LC_75_ were estimated by using Ecanything from LC_50_ (https://www.graphpad.com/quickcalcs/Ecanything1.cfm (accessed on 5 March 2026), Quick Calcs—GraphPad; EC: effective concentration) and entering the LC_50_ HillSlope of *P. hemmendorffii* EO or limonene. Data distribution and normality were tested using the Kolmogorov–Smirnov test (*p* > 0.05). Comparisons between groups were done using a one-way ANOVA followed by Tukey’s multiple comparison Test (*p* < 0.05).

## 3. Results

### 3.1. Analysis of Essential Oil Composition

The EO of *P. hemmendorffii* was analyzed by gas chromatography–mass spectrometry (GC–MS) to determine its main constituents. Compound identification was based on mass spectral library matching, a comparison of retention indices (RI), and, when available, co-injection with authentic standards. The relative percentages of each constituent are presented in Table 1. A total of forty-one compounds were identified in the EO, which were classified as monoterpene hydrocarbons, sesquiterpenes, oxygenated monoterpenes, and oxygenated sesquiterpenes. The major compound in the EO, as determined by GC–MS analysis, was limonene (30.9%) (Table 1), which was subsequently selected for use in the bioassays.

### 3.2. Effect of P. hemmendorffii EO and Limonene on Larval Mortality

*Piper hemmendorffii* EO exhibited higher larvicidal activity (LC_50_ = 10.46 mg/mL; 95% CI = 9.86 to 11.09) compared to limonene (LC_50_ = 31.62 mg/mL; 95% CI = 30.86 to 32.40). The difference between treatments was statistically supported by non-overlapping confidence intervals. The lethal concentrations (LC_5_, LC_25_, LC_50_, and LC_75_) of the compounds against *R. microplus* larvae are presented in Table 2.

### 3.3. Effect of P. hemmendorffii EO and Limonene on Detoxifying Enzymes of Larvae

**Figure 1:**
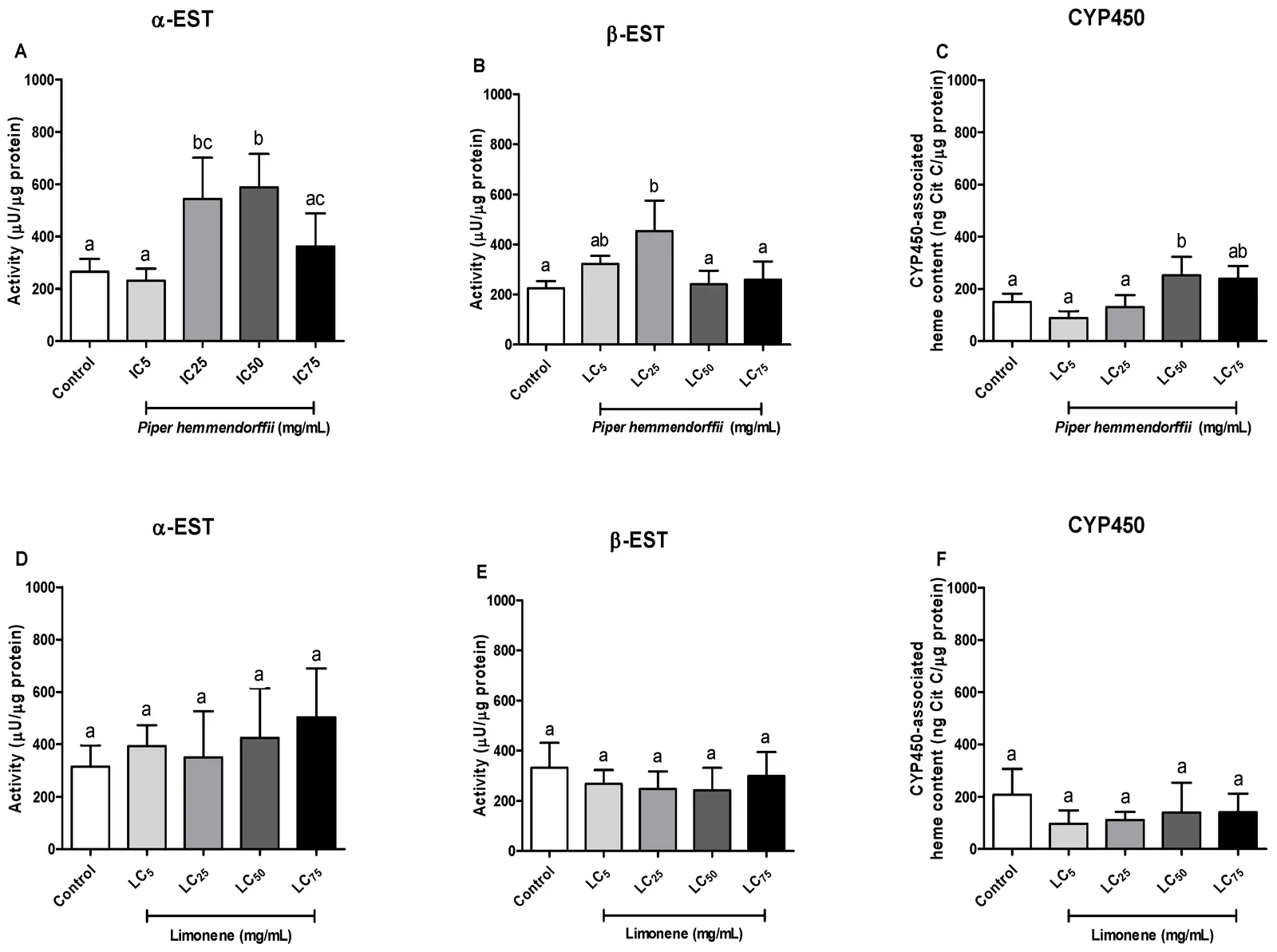
Effect of *Piper hemmendorffii* EO (**A**–**C**) and limonene (**D**–**F**) on enzymatic activities of α-esterase (α-EST), β-esterase (β-EST) and CYP450-associated heme/mixed-function oxidase content (CYP450) of *Rhipicephalus microplus* larvae. Means with different letters are significantly different from each other (*p* < 0.05 as determined by one-way ANOVA followed by Tukey’s post hoc test). Bars represent mean ± standard deviation of five biological replicates as determined by one-way ANOVA followed by Dunnett’s multiple comparison.

Regarding α-EST activity, the lowest concentration of *P. hemmendorffii* EO (LC_5_; 230.7 ± 46.29 µU/µg) did not significantly affect enzyme activity compared to the control group (265 ± 49.58 µU/µg) (*p* > 0.05). In contrast, LC_25_ (543 ± 157.5 µU/µg) and LC_50_ (587.8 ± 127.2 µU/µg) induced a significant increase in α-EST activity (F (4, 20) = 10.56, *p* < 0.0001), with the highest mean values observed at LC_50_. In contrast, LC_75_ exhibited an effect (α-EST: 361.6 ± 126.4 µU/µg), showing no significant difference (*p* > 0.05) compared to the control, LC_5_ and LC_25_. Regarding β-EST activity, *P. hemmendorffii* EO induced a significant increase (F (4, 20) = 8.73, *p* < 0.001) only at LC_25_ (β-EST: 453 ± 121.6 µU/µg). This treatment differed significantly from the control group (β-EST: 225 ± 28.21 µU/µg) (*p* < 0.05) (and from the higher concentrations, LC_50_ (β-EST: 241.5 ± 53.30 µU/µg) (*p* < 0.05) and LC_75_ (β-EST: 259.7 ± 72.82 µU/µg) (*p* < 0.05)) (Figure 1).

For the *P. hemmendorffii* EO, CYP450-associated heme/mixed-function oxidase content did not show a significant difference compared to the control group (CYP450: 149.3 ± 31.68 ng CytC/µg protein) at LC_5_ (CYP450: 87.25 ± 27.03 ng CytC/µg protein) and LC_25_ (CYP450: 129.5 ± 45.61 ng CytC/µg protein) but increased significantly (F (4, 20) = 11.33, *p* < 0.0001) at LC_50_ (CYP450: 251 ± 70.57 ng CytC/µg protein) (Figure 1).

The exposure of larvae to the four lethal concentrations of limonene did not result in significant alterations in the activities of α-EST (F (4, 20) = 1.83, *p* = 0.16), β-EST (F (4, 20) = 1, *p* = 0.42), or CYP450 (F (4, 20) = 1.42, *p* = 0.26) compared with the control group. Although minor differences in mean enzymatic activity were observed among treatments, they were not statistically significant, indicating no enzymatic induction or inhibition under the experimental conditions (Figure 1).

### 3.4. Effect of Piper hemmendorffii EO and Limonene on Acetylcholinesterase (AChE) Activity of Larvae

**Figure 2:**
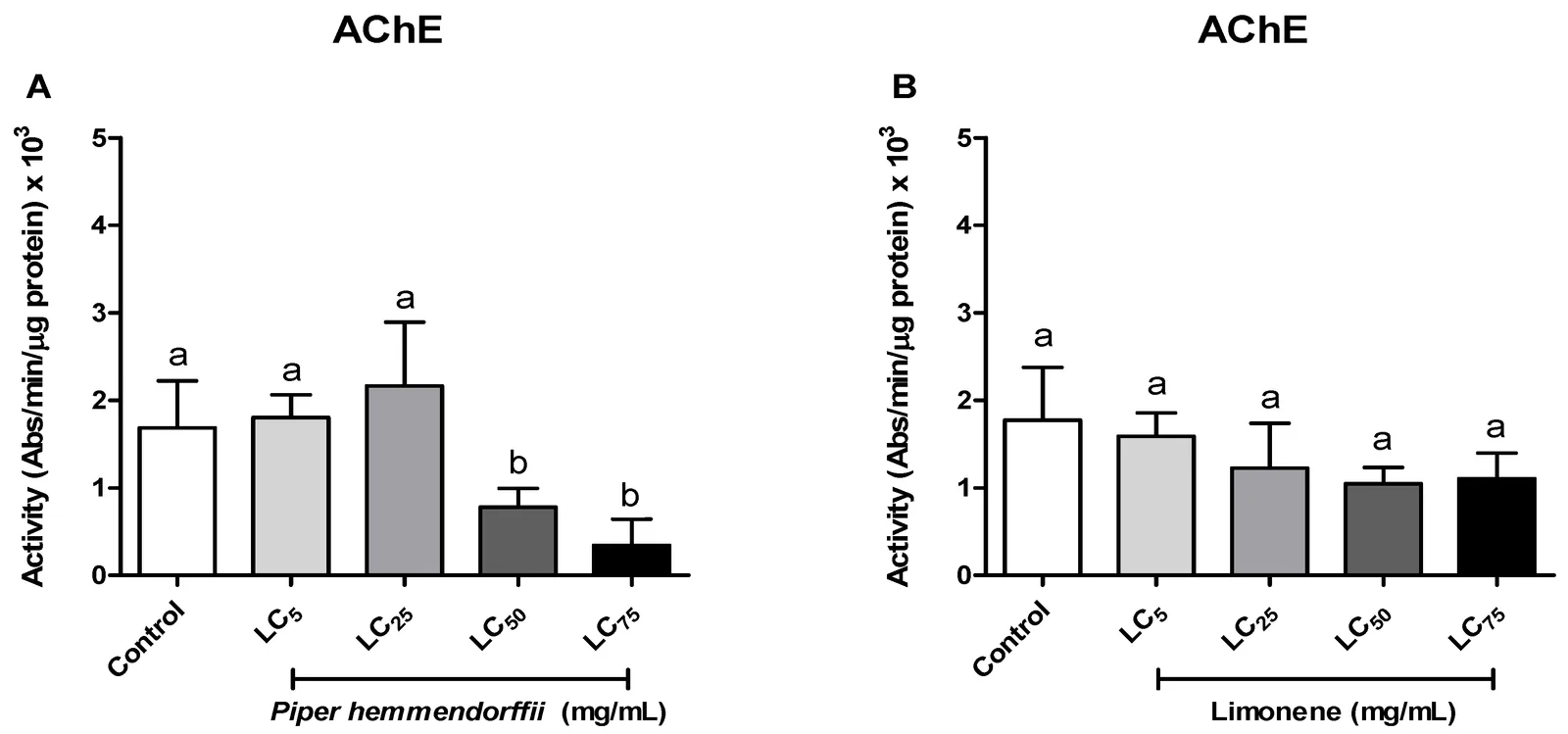
Effect of *Piper hemmendorffii* EO (**A**) and limonene (**B**) on acetylcholinesterase (AChE) activity of *Rhipicephalus microplus* larvae. Means with different letters are significantly different from each other (*p* < 0.05) in ANOVA followed by Tukey’s multiple comparison. Bars represent mean ± standard deviation (SD) of five biological replicates.

The effects of *P. hemmendorffii* EO and its major constituent, limonene, on the AChE activity of *R. microplus* larvae were evaluated under the conditions described in Section 2.4.4. The exposure of *R. microplus* larvae to *P. hemmendorffii* EO resulted in a significant reduction in AChE activity at LC_50_ (10.46 mg/mL; 0.77 ± 0.21 Abs/min/μg protein × 10^3^) and LC_75_ (14.96 mg/mL; 0.34 ± 0.29 Abs/min/μg protein × 10^3^) compared with the control group (1.65 ± 0.53 Abs/min/μg protein × 10^3^) (F (4, 20) = 14.21, *p* < 0.0001). Although the overall one-way ANOVA indicated a significant difference among treatments (F(4,20) = 3.09, *p* = 0.039), none of the treatment groups differed significantly from the control in the subsequent Tukey multiple comparison test (*p* > 0.05 for all comparisons) (Figure 2).

### 3.5. Scanning Electron Microscopy

**Figure 3:**
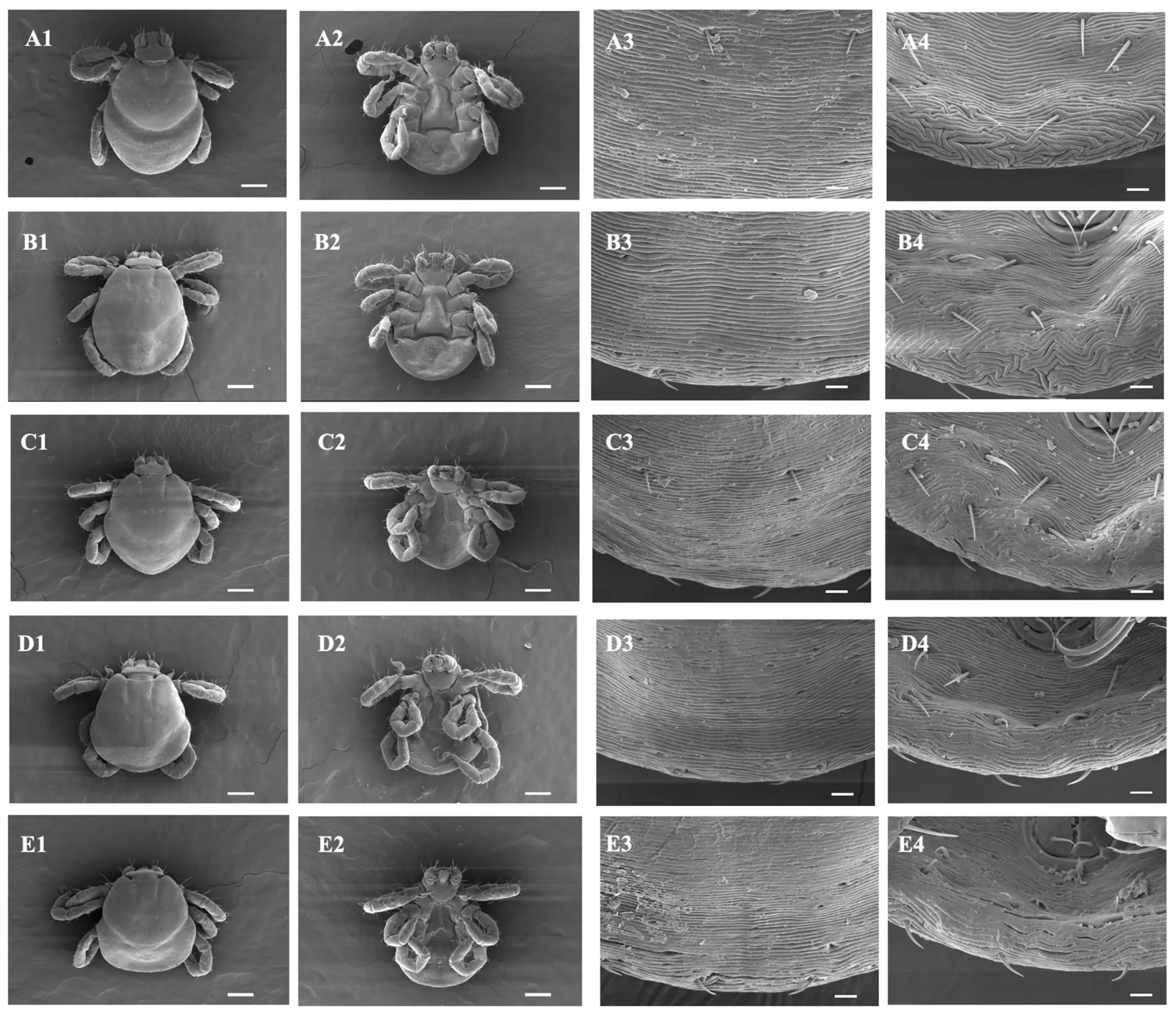
Scanning electron microscopy images illustrating the cuticle of *Rhipicephalus microplus* larvae after exposure to the different experimental treatments. Vehicle control (1% acetone and 0.02% Triton X-100)—(**A1**) (dorsal view × 120), (**A2**) (ventral view × 120), (**A3**) (dorsal view × 1000) and (**A4**) (ventral view × 1000); *Piper hemmendorffii* (LC_50_ mg/mL)—(**B1**) (dorsal view × 120), (**B2**) (ventral view × 120), (**B3**) (dorsal view × 1000) and (**B4**) (ventral view × 1000); *Piper hemmendorffii* (LC_75_ mg/mL)—(**C1**) (dorsal view × 120), (**C2**) (ventral view × 120), (**C3**) (dorsal view × 1000) and (**C4**) (ventral view × 1000); limonene (LC_50_ mg/mL)—(**D1**) (dorsal view × 120), (**D2**) (ventral view × 120), (**D3**) (dorsal view × 1000) and (**D4**) (ventral view × 1000); limonene (LC_75_ mg/mL)—(**E1**) (dorsal view × 120), (**E2**) (ventral view × 120), (**E3**) (dorsal view × 1000) and (**E4**) (ventral view × 1000). (**A1**,**A2**,**B1**,**B2**,**C1**,**C2**,**D1**,**D2**,**E1**,**E2**) scale bars = 100 µm and (**A3**,**A4**,**B3**,**B4**,**C3**,**C4**,**D3**,**D4**,**E3**,**E4**) scale bars = 10 µm.

A total of ten images were acquired for each experimental group, consisting of five dorsal and five ventral views, according to the methodology described in Section 2.5. Scanning electron microscopy did not demonstrate detectable changes in the cuticular surface of *R. microplus* larvae after in vitro exposure to *P. hemmendorffii* and limonene at LC_50_ and LC_75_ concentrations (Figure 3). The cuticle remained morphologically preserved, without evidence of structural disorganization, fissuring, or deposition, and was indistinguishable from that of the vehicle control group.

## 4. Discussion

Although synthetic chemical agents have played a central role in tick control, their extensive and continuous application has raised significant concerns regarding environmental safety and public health while also contributing to the widespread development of resistance among tick populations [32,33]. Given these limitations, increasing interest has been directed toward plant-based approaches for tick control. Although the LC_50_ value obtained for *P. hemmendorffii* EO (10.46 mg/mL) indicates acaricidal potential under laboratory conditions, its practical application in the field requires further evaluation. Factors such as formulation stability, residual activity, environmental persistence, and production costs may influence its feasibility. Compared with botanical acaricides such as azadirachtin [34] and carvacrol [35], which show variable efficacy depending on formulation and exposure conditions, the present results suggest that *P. hemmendorffii* EO is a promising candidate requiring additional formulation studies and field validation before practical use.

The genus *Piper* has been recognized as a potential source of bioactive metabolites relevant for tick control strategies, such as *P. nigrum* [36] and *P. aduncum* [11] against *R. microplus*. In the present study, we report for the first time the effects of *P. hemmendorffii* EO and its major constituent, limonene, on the activity of detoxification enzymes in *R. microplus* larvae, providing new insights into the metabolic responses associated with exposure to these natural products. Based on the GC–MS chemical characterization, limonene was identified as the major constituent of the EO of *P. hemmendorffii* (30.9%) and was chosen for evaluation. The evaluation of the biological activity of the major compound identified in an EO is important because the activity of the whole EO may also be influenced by the presence and relative abundance of other constituents.

Limonene exhibited lower activity (LC_50_ = 31.62 mg/mL; 95% CI = 30.86–32.40) in comparison to *P. hemmendorffii* EO (LC_50_ = 10.46 mg/mL; 95% CI = 9.86–11.09), with a significant difference observed between the groups, based on non-overlapping confidence intervals. The lower acaricidal activity observed for limonene when tested individually indicates that its effect is considerably weaker in isolation. This pattern has been previously reported in studies with *R. microplus*, where EOs showed greater acaricidal activity than their purified constituents, suggesting that other constituents present in the EO may contribute to its overall acaricidal activity [37] and suggesting the involvement of different physiological pathways in *R. microplus* larvae. Besides limonene, the EO contains relevant amounts of β-pinene (10.1%) and β-caryophyllene (9.6%), which have been reported to exhibit bioactive properties against ticks [38,39]. The contribution of these and other constituents may help explain the enhanced acaricidal effect observed for the EO; however, further studies with isolated compounds and defined mixtures are needed to determine their individual contributions and possible interactions.

In the development of new acaricidal products, monitoring the impact of new substances on detoxification enzymes in ticks is crucial, as alterations in enzymatic activity may indicate the induction of metabolic resistance and provide early insight into the potential effectiveness of these compounds [40,41]. In this context, this study provides the first evidence that *P. hemmendorffii* can modulate key detoxification and neurophysiological targets in *R. microplus*. The effects were observed on enzymatic systems involved in xenobiotic metabolism, including α-EST, β-EST, and CYP450, as well as on AChE, an essential enzyme for nervous system function.

In the EO of *P. hemmendorffii*, α-EST activity increased significantly at LC_25_ and LC_50_, peaking at LC_50_, while LC_5_ and LC_75_ did not differ from the control. In contrast, β-EST activity increased only at LC_25_, returning to control levels at higher concentrations. The observed increase in esterase activity may reflect a physiological adjustment to chemical stress, a phenomenon frequently described in acaricide-exposed *R. microplus*, where elevated enzymatic activity constitutes an important metabolic barrier against xenobiotic toxicity [42,43,44]. CYP450-associated heme/mixed-function oxidase content was significantly elevated at LC_50_, with a secondary, intermediate response at LC_75_, suggesting the modulation of oxidative biotransformation processes involved in xenobiotic metabolism at these exposure levels. Together, these results suggest changes in EST and CYP450-associated heme/mixed-function oxidase content that may be associated with altered detoxification capacity. However, because the biochemical analyses were conducted only on larvae surviving 24 h of exposure, these enzymatic profiles may reflect, at least in part, the selection of surviving individuals rather than treatment-induced responses representative of the entire exposed population. Therefore, the observed changes should be interpreted cautiously and do not by themselves demonstrate the induction or activation of detoxification pathways. The increase in EST and CYP450-associated heme/mixed-function oxidase content likely reflects an adaptive physiological response or the onset of metabolic tolerance rather than a direct cause of mortality, indicating that enhanced detoxification capacity alone is insufficient to prevent toxic effects at higher concentrations. It is important to consider that the *R. microplus* population evaluated in this study is resistant to cypermethrin (resistance level I), which may influence the basal activity of detoxification pathways. Resistant populations may exhibit alterations in metabolic enzymes, including esterases and cytochrome P450 monooxygenases, contributing to acaricide tolerance. Therefore, the enzymatic responses observed after exposure to *P. hemmendorffii* EO should be interpreted considering this resistant background. Future studies comparing susceptible and resistant strains will be important to distinguish constitutive resistance mechanisms from compound-induced enzymatic modulation.

In contrast to the results observed for the EO of *P. hemmendorffii*, *R. microplus* larvae exposed to limonene showed no modulation of the three detoxification enzymes evaluated, indicating that these metabolic pathways were not affected under the experimental conditions tested. Another point that justifies consideration is that this limited enzymatic response may be related to the relatively low acaricidal activity of limonene, resulting in a reduced impact on tick metabolism [6,45]. Two hypotheses may explain the present findings: First, the lack of alteration in detoxification enzymes in *R. microplus* suggests that limonene may be processed through basal metabolic pathways, possibly involving biotransformation similar to that observed in *Spodoptera litura* Fabricius, 1775 larvae, without inducing an adaptive enzymatic response [46]. Second, limonene toxicity may involve oxidative stress pathways, since monoterpenes can interfere with antioxidant enzymes such as catalase (CAT), superoxide dismutase (SOD), and glutathione peroxidase (GPx) in ticks, which are important for maintaining cellular homeostasis and viability [47]. However, terpene metabolism and the involvement of detoxification enzymes are highly species-dependent among arthropods. Insect species possess diverse detoxification systems, including cytochrome P450 monooxygenases and glutathione S-transferases, which may respond differently depending on the compound and biological context [48]. Thus, further studies are required to investigate these hypotheses and to improve our understanding of the relationship between emerging compounds and specific enzymatic systems.

The EO of *P. hemmendorffii* significantly reduced AChE activity in *R. microplus* larvae at LC_50_ and LC_75_ concentrations, indicating that the impairment of cholinergic neurotransmission may contribute to its acaricidal activity. Similar AChE inhibitory effects have been reported for terpenoid-rich EOs against *R. microplus*, reinforcing the role of neurotoxicity as an important mechanism of action of plant-derived acaricides [49]. In contrast, limonene did not affect enzymatic activity, indicating that the inhibitory effect of the EO cannot be attributed to its major compound alone and may be associated with the presence of other constituents, although their individual or combined contributions were not experimentally evaluated. Among these constituents, β-pinene is a plausible candidate for contributing to the observed activity because of its previously reported acaricidal effects against *R. microplus* [50]. Nevertheless, its individual contribution was not evaluated in the present study.

The enzymatic assays indicate that the EO of *P. hemmendorffii* interferes with detoxification and neural signaling enzymes in *R. microplus* larvae. In contrast, scanning electron microscopy revealed no detectable cuticular alterations after exposure to the EO or its major compound, suggesting that their acaricidal activity is not primarily associated with damage to external tick structures. Similar findings have been reported for *P. aduncum* EO, which also did not promote cuticular disruption in ticks, including another species from the family Ixodidae, *Amblyomma sculptum* Berlese, 1888 [51]. Further investigations involving internal tissue analyses are therefore needed to clarify the mechanisms underlying the toxicity of these compounds in different tick organs such as the synganglion and salivary glands [52].

## 5. Conclusions

The findings indicate that *P. hemmendorffii* EO is a promising natural acaricidal agent against *R. microplus* larvae, with its superior efficacy likely reflecting the contribution of other constituents or possible interactions among them, rather than that of limonene alone. The observed enzymatic responses provide valuable insights into potential mechanisms underlying its biological activity and support the further investigation of its mode of action. Future studies should focus on histological analyses to identify the primary cellular targets of the EO and explore the potential of limonene in combination with synthetic acaricides to develop more effective and sustainable tick control strategies.

## Figures and Tables

**Table 1:** Chemical composition of essential oil of *Piper hemmendorffii*.

Compounds	RI ^a^	RI ^b^	%
α-Pinene	932	932	3.2
β-Pinene	975	974	10.1
β-Myrcene	992	988	0.7
2-Carene	1010	1008	0.5
*p*-Cymene	1024	1020	0.3
Limonene	1039	1024	30.9
(Z)-β-Ocimene	1039	1032	0.3
(E)-β-Ocimene	1049	1044	3.2
α-Terpinolene	1088	1086	0.1
(E)-4.8-Dimethyl-1.3.7-nonatriene (DMNT)	1117	1114	0.6
α-Terpineol	1191	1186	0.7
δ-Elemene	1339	1335	0.1
α-Cubebene	1352	1345	0.3
α-Ylangene	1374	1373	0.1
α-Copaene	1378	1374	4.1
β-Elemene	1394	1389	0.6
(Z)-Caryophyllene	1404	1408	0.5
α-Gurjunene	1412	1409	0.2
€-β-Caryophyllene	1422	1417	9.6
β-Gurjunene	1432	1431	0.9
(+)-Aromadendrene	1442	1439	0.4
α-Humulene	1457	1452	2.7
(–)-Alloaromadendrene	1464	1458	0.5
γ-Muurolene	1480	1479	1.3
Germacrene D	1484	1481	2.7
β-Selinene	1490	1490	1.0
α-Selinene	1500	1498	1.7
Bicyclogermacrene	1500	1500	1.7
α-Muurolene	1503	1500	0.9
γ-Cadinene	1517	1522	1.3
(E)-Cadina-1.4-diene	1527	1533	7.1
Germacrene B	1561	1559	0.6
(E)-Nerolidol	1566	1561	0.9
Palustrol	1572	1567	0.3
Spathulenol	1581	1578	0.8
Caryophyllene oxide	1587	1582	4.3
Viridiflorol	1596	1592	0.3
Humulene epoxide II	1613	1608	0.3
epi-α-Muurolol	1646	1640	1.1
Torreyol	1650	1644	1.1
α-Cadinol	1659	1652	1.7

RI ^a^: The retention index calculated against C8-C40 n-alkanes using an HP-5 m column. RI ^b^: Retention index values from the literature (Adams, 2007) [31]. Compound identity confirmed with an authentic standard. The remaining compounds were identified by comparing the retention indices and mass spectra with the Adams and Wiley spectral databases (see text for details).

**Table 2:** Lethal concentrations (LC–mg/mL) of *Piper hemmendorffii* and limonene against *Rhipicephalus microplus* larvae.

EO/Compound	LC_5_ *	LC_25_ *	LC_50_	LC_75_ *	HillSlope ± SE	CI 95%	R^2^
*Piper hemmendorffii*	4.00	7.31	10.46	14.96	3.06 ± 0.27	9.86 to 11.09	0.86
Limonene	20.11	26.71	31.62	37.42	6.51 ± 0.46	30.86 to 32.40	0.92

* LC_5_, LC_25_ and LC_75_ were estimated from EC from EC_50_ (Quick Calcs—GraphPad) and the HillSlope value of LC_50_ for each treatment. LC_50_: lethal concentration (mg/mL) for 50% of individuals; SE: standard error; CI 95%: 95% confidence interval; R^2^: coefficient of determination. All experiments were conducted in duplicates (technical replicates) and repeated in five independent assays (biological replicates). Each technical replicate included 100–150 larvae.

## Data Availability

Data are available in the manuscript.

## References

[1] Cruz B.C., De Lima Mendes A.F., Maciel W.G., Dos Santos I.B., Gomes L.V.C., Felippelli G., Teixeira W.F.P., Ferreira L.L., Soares V.E., Lopes W.D.Z. (2020). Biological Parameters for *Rhipicephalus microplus* in the Field and Laboratory and Estimation of Its Annual Number of Generations in a Tropical Region. Parasitol. Res..

[2] Sales D.P., Silva-Junior M.H.S., Tavares C.P., Sousa I.C., Sousa D.M., Brito D.R.B., Camargo A.M., Leite R.C., Faccini J.L.H., Lopes W.D.Z. (2024). Biology of the Non-Parasitic Phase of the Cattle Tick *Rhipicephalus (Boophilus) microplus* in an Area of Amazon Influence. Parasit. Vectors.

[3] Addo S.O., Bentil R.E., Baako B.O.A., Addae C.A., Larbi J.A., Baidoo P.K., Wilson M.D., Asoala V., Oduro D., Mate S. (2023). First Record of *Rhipicephalus (Boophilus) microplus* in Ghana, a Potential Risk to Livestock Production. Exp. Appl. Acarol..

[4] Rodriguez-Vivas R.I., Jonsson N.N., Bhushan C. (2018). Strategies for the Control of *Rhipicephalus microplus* Ticks in a World of Conventional Acaricide and Macrocyclic Lactone Resistance. Parasitol. Res..

[5] Gonzaga B.C.F., Barrozo M.M., Coutinho A.L., Pereira e Sousa L.J.M., Vale F.L., Marreto L., Marchesini P., de Castro Rodrigues D., de Souza E.D.F., Sabatini G.A. (2023). Essential Oils and Isolated Compounds for Tick Control: Advances beyond the Laboratory. Parasit. Vectors.

[6] De Assis Lage T.C., Montanari R.M., Fernandes S.A., De Oliveira Monteiro C.M., De Oliveira Souza Senra T., Zeringota V., Da Silva Matos R., Daemon E. (2015). Chemical Composition and Acaricidal Activity of the Essential Oil of *Baccharis dracunculifolia* De Candole (1836) and Its Constituents Nerolidol and Limonene on Larvae and Engorged Females of *Rhipicephalus microplus* (Acari: Ixodidae). Exp. Parasitol..

[7] de Assis Lage T.C., Montanari R.M., Fernandes S.A., de Oliveira Monteiro C.M., de Oliveira Souza Senra T., Zeringota V., Calmon F., da Silva Matos R., Daemon E. (2013). Activity of Essential Oil of *Lippia triplinervis* Gardner (Verbenaceae) on *Rhipicephalus microplus* (Acari: Ixodidae). Parasitol. Res..

[8] Ferreira F.M., Delmonte C.C., Novato T.L.P., Monteiro C.M.O., Daemon E., Vilela F.M.P., Amaral M.P.H. (2018). Acaricidal Activity of Essential Oil of *Syzygium aromaticum*, Hydrolate and Eugenol Formulated or Free on Larvae and Engorged Females of *Rhipicephalus microplus*. Med. Vet. Entomol..

[9] Da Silva J., Da Trindade R., Alves N., Figueiredo P., Maia J., Setzer W. (2017). Essential Oils from Neotropical Piper Species and Their Biological Activities. Int. J. Mol. Sci..

[10] Salehi B., Zakaria Z.A., Gyawali R., Ibrahim S.A., Rajkovic J., Shinwari Z.K., Khan T., Sharifi-Rad J., Ozleyen A., Turkdonmez E. (2019). Piper Species: A Comprehensive Review on Their Phytochemistry, Biological Activities and Applications. Molecules.

[11] Silva W.C., De Souza Martins J.R., De Souza H.E.M., Heinzen H., Cesio M.V., Mato M., Albrecht F., De Azevedo J.L., De Barros N.M. (2009). Toxicity of *Piper aduncum* L. (Piperales: Piperaceae) from the Amazon Forest for the Cattle Tick *Rhipicephalus (Boophilus) microplus* (Acari: Ixodidae). Vet. Parasitol..

[12] Da Silva Lima A., Do Nascimento Sousa Filho J.G., Garcia Pereira S., Skelding Pinheiro Guillon G.M., Da Silva Santos L., Costa Júnior L.M. (2014). Acaricide Activity of Different Extracts from *Piper tuberculatum* Fruits against *Rhipicephalus microplus*. Parasitol. Res..

[13] Godara R., Verma M.K., Katoch R., Yadav A., Dutt P., Satti N.K., Katoch M. (2018). In Vitro Acaricidal Activity of Piper Nigrum and Piper Longum Fruit Extracts and Their Active Components against *Rhipicephalus (Boophilus) microplus* Ticks. Exp. Appl. Acarol..

[14] Flora & Funga do Brasil. Jardim Botânico Do Rio de Janeiro. http://Floradobrasil.Jbrj.Gov.Br/.

[15] Govaerts R. World Checklist of Vascular Plants (WCVP)—Version 12. 2023. Facilitated by the Royal Botanic Gardens, Kew. http://wcvp.science.kew.org/.

[16] Bass C., Field L.M. (2011). Gene Amplification and Insecticide Resistance. Pest. Manag. Sci..

[17] Li X., Schuler M.A., Berenbaum M.R. (2007). Molecular Mechanisms of Metabolic Resistance to Synthetic and Natural Xenobiotics. Annu. Rev. Entomol..

[18] Lahiri R., Kumar R., Arya S., Singh A.P., Negi S., Joshi S., Bargali P., Mali S.N., Rawat S., de Oliveira M.S. (2026). Bioactivity Profiling of Coronarin A and Villosin Isolated From *Hedychium coronarium* J. Koenig: Acaricidal, Nematicidal, and Antifungal Potential. Chem. Biodivers..

[19] Ibrahium S.M., Abdel-Baki A.-A.S., Abo El-Ela F.I., Balegh A.A., Wahba A.A., Al-Quraishy S., Awad E.M., Ahmed M., Mahmoud W.G., Aboelhadid S.M. (2026). In Vitro and In Silico Investigations of the Acaricidal Activity of Carvacrol Against *Rhipicephalus annulatus*. Acta Parasitol..

[20] Hernandez R., He H., Chen A.C., Ivie G.W., George J.E., Wagner G.G. (1999). Cloning and Sequencing of a Putative Acetylcholinesterase cDNA from *Boophilus microplus* (Acari: Ixodidae). J. Med. Entomol..

[21] Coles T.B., Dryden M.W. (2014). Insecticide/Acaricide Resistance in Fleas and Ticks Infesting Dogs and Cats. Parasit. Vectors.

[22] Aziz Z.A.A., Ahmad A., Setapar S.H.M., Karakucuk A., Azim M.M., Lokhat D., Rafatullah M., Ganash M., Kamal M.A., Ashraf G.M. (2018). Essential Oils: Extraction Techniques, Pharmaceutical and Therapeutic Potential—A Review. Curr. Drug Metab..

[23] Fanela T.L.M., Baldin E.L.L., Pannuti L.E.R., Cruz P.L., Crotti A.E.M., Takeara R., Kato M.J. (2016). Lethal and Inhibitory Activities of Plant-Derived Essential Oils Against *Bemisia tabaci* Gennadius (Hemiptera: Aleyrodidae) Biotype B in Tomato. Neotrop. Entomol..

[24] Pereira Filho A.A., Pessoa G.C.D., Yamaguchi L.F., Stanton M.A., Serravite A.M., Pereira R.H.M., Neves W.S., Kato M.J. (2021). Larvicidal Activity of Essential Oils From Piper Species Against Strains of *Aedes aegypti* (Diptera: Culicidae) Resistant to Pyrethroids. Front. Plant Sci..

[25] Food and Agriculture Organization of the United Nations (2025). Guidelines for Sustainable Tick Control and Acaricide Resistance Mitigation in Livestock.

[26] Zaldivar M.F., Bastianetto E., Pereira Filho A.A., Rodrigues D.S., Martins Júnior V.S., Morais-Costa F., Vasconcelos V.O., Duarte E.R., Araujo R.N. (2024). Acaricide Effect of Plants from the Brazilian Savanna on a Population of *Rhipicephalus microplus* with Phenotypic Resistance to Cypermethrin and Trichlorfon. Vet. Parasitol..

[27] Klafke G.M., Sabatini G.A., De Albuquerque T.A., Martins J.R., Kemp D.H., Miller R.J., Schumaker T.T.S. (2006). Larval Immersion Tests with Ivermectin in Populations of the Cattle Tick *Rhipicephalus* (*Boophilus*) *microplus* (Acari: Ixodidae) from State of Sao Paulo, Brazil. Vet. Parasitol..

[28] Adhikari K., Khanikor B., Sarma R. (2022). Persistent Susceptibility of *Aedes aegypti* to Eugenol. Sci. Rep..

[29] Van Asperen K. (1962). A Study of Housefly Esterases by Means of a Sensitive Colorimetric Method. J. Insect Physiol..

[30] Li A.Y., Pruett J.H., Davey R.B., George J.E. (2005). Toxicological and Biochemical Characterization of Coumaphos Resistance in the San Roman Strain of *Boophilus microplus* (Acari: Ixodidae). Pestic. Biochem. Physiol..

[31] Adams R.P. (2007). Identification of Essential Oil Components by Gas Chromatography Mass Spectroscopy.

[32] Cheng J. (2026). Dynamics of a Tick-Borne Disease Transmission Model with Acquired Tick Resistance. Math. Biosci..

[33] Rodriguez–Vivas R.I., Flota-Burgos G.J., Torres-Castro M., Sabatini G.A. (2026). Factors Influencing the Selection of *Rhipicephalus microplus* Resistance to Acaricides. Vet. Parasitol..

[34] Giglioti R., Forim M.R., Oliveira H.N., Chagas A.C.S., Ferrezini J., Brito L.G., Falcoski T.O.R.S., Albuquerque L.G., Oliveira M.C.S. (2011). In Vitro Acaricidal Activity of Neem (*Azadirachta indica*) Seed Extracts with Known Azadirachtin Concentrations against *Rhipicephalus microplus*. Vet. Parasitol..

[35] Novato T., Gomes G.A., Zeringóta V., Franco C.T., de Oliveira D.R., Melo D., de Carvalho M.G., Daemon E., de Oliveira Monteiro C.M. (2018). In Vitro Assessment of the Acaricidal Activity of Carvacrol, Thymol, Eugenol and Their Acetylated Derivatives on *Rhipicephalus microplus* (Acari: Ixodidae). Vet. Parasitol..

[36] Vinturelle R., Mattos C., Meloni J., Nogueira J., Nunes M.J., Vaz I.S., Rocha L., Lione V., Castro H.C., Chagas E.F.D. (2017). In Vitro Evaluation of Essential Oils Derived from *Piper nigrum* (Piperaceae) and *Citrus limonum* (Rutaceae) against the Tick *Rhipicephalus (Boophilus) microplus* (Acari: Ixodidae). Biochem. Res. Int..

[37] Martinez-Velazquez M., Castillo-Herrera G.A., Rosario-Cruz R., Flores-Fernandez J.M., Lopez-Ramirez J., Hernandez-Gutierrez R., Del Carmen Lugo-Cervantes E. (2011). Acaricidal Effect and Chemical Composition of Essential Oils Extracted from *Cuminum cyminum*, *Pimenta dioica* and *Ocimum basilicum* against the Cattle Tick *Rhipicephalus (Boophilus) microplus* (Acari: Ixodidae). Parasitol. Res..

[38] Arantes A.C.S., Ribeiro J.C.S., Soares D.S., Reis A.C., das Graças Cardoso M., Remedio R.N. (2024). Alpha- and Beta-Pinene Isomers Act Differently to Control *Rhipicephalus microplus* (Acari: Ixodidae). Parasitol. Res..

[39] Chigure G.M., Sharma A.K., Ajithkumar K.G., Fular A., Tayade A.B., Kumar R., Jadhav N.D., Kumar S., Gupta S., Nagar G. (2025). Evaluation of *β-Caryophyllene(1–3)* as a Potential Anti-Tick Molecule for Controlling Tick Infestations on Animals. Exp. Parasitol..

[40] De Rouck S., İnak E., Dermauw W., Van Leeuwen T. (2023). A Review of the Molecular Mechanisms of Acaricide Resistance in Mites and Ticks. Insect Biochem. Mol. Biol..

[41] Obaid M.K., Islam N., Alouffi A., Khan A.Z., da Silva Vaz I., Tanaka T., Ali A. (2022). Acaricides Resistance in Ticks: Selection, Diagnosis, Mechanisms, and Mitigation. Front. Cell. Infect. Microbiol..

[42] Baffi M.A., De Souza G.R.L., De Sousa C.S., Ceron C.R., Bonetti A.M. (2008). Esterase Enzymes Involved in Pyrethroid and Organophosphate Resistance in a Brazilian Population of *Riphicephallus* (*Boophilus*) *microplus* (Acari, Ixodidae). Mol. Biochem. Parasitol..

[43] Gaur R.S., Sangwan A.K., Sangwan N., Ghosh M., Kumar S. (2017). Comparative Study of Esterases in Deltamethrin and Diazinon Resistant *Rhipicephalus microplus* and *Hyalomma anatolicum* Ticks Collected from the Trans-Gangetic Plains of India. Exp. Appl. Acarol..

[44] Singh N.K., Rath S.S. (2014). Esterase Mediated Resistance against Synthetic Pyrethroids in Field Populations of *Rhipicephalus (Boophilus) microplus* (Acari: Ixodidae) in Punjab Districts of India. Vet. Parasitol..

[45] Jyoti, Manisha, Singh H., Singh N.K. (2025). Acaricidal Activity of Various Essential Oil Components against Acaricide-Resistant *Rhipicephalus microplus* (Acari: Ixodidae). Exp. Parasitol..

[46] Miyazawa M., Wada T., Kameoka H. (1998). Biotransformation of (+)- and (−)-Limonene by the Larvae of Common Cutworm (*Spodoptera litura*). J. Agric. Food Chem..

[47] Tavares C.P., Sabadin G.A., Sousa I.C., Gomes M.N., Soares A.M.S., Monteiro C.M.O., Vaz I.S., Costa-Junior L.M. (2022). Effects of Carvacrol and Thymol on the Antioxidant and Detoxifying Enzymes of *Rhipicephalus microplus* (Acari: Ixodidae). Ticks Tick-Borne Dis..

[48] Hilliou F., Chertemps T., Maïbèche M., Le Goff G. (2021). Resistance in the Genus Spodoptera: Key Insect Detoxification Genes. Insects.

[49] Cardoso A.D.S., Santos E.G.G., Lima A.D.S., Temeyer K.B., Pérez De León A.A., Costa L.M., Soares A.M.D.S. (2020). Terpenes on *Rhipicephalus* (*Boophilus*) *microplus*: Acaricidal Activity and Acetylcholinesterase Inhibition. Vet. Parasitol..

[50] Santos E.G.G.D., Bezerra W.A.D.S., Temeyer K.B., León A.A.P.D., Costa-Junior L.M., Soares A.M.D.S. (2021). Effects of Essential Oils on Native and Recombinant Acetylcholinesterases of *Rhipicephalus microplus*. Rev. Bras. Parasitol. Veterinária.

[51] Pereira Filho A.A., Do Vale V.F., De Oliveira Monteiro C.M., Barrozo M.M., Stanton M.A., Yamaguchi L.F., Kato M.J., Araújo R.N. (2024). Effects of *Piper aduncum* (Piperales: Piperaceae) Essential Oil and Its Main Component Dillapiole on Detoxifying Enzymes and Acetylcholinesterase Activity of *Amblyomma sculptum* (Acari: Ixodidae). Int. J. Mol. Sci..

[52] Matos R.S., Daemon E., De Oliveira Monteiro C.M., Sampieri B.R., Marchesini P.B.C., Delmonte C., Camargo-Mathias M.I. (2019). Thymol Action on Cells and Tissues of the Synganglia and Salivary Glands of *Rhipicephalus sanguineus* Sensu Lato Females (Acari: Ixodidae). Ticks Tick-Borne Dis..

